# The prognostic significance of Flap Endonuclease 1 (FEN1) in breast ductal carcinoma in situ

**DOI:** 10.1007/s10549-021-06271-y

**Published:** 2021-06-12

**Authors:** Abdulbaqi Al-Kawaz, Islam M. Miligy, Michael S. Toss, Omar J. Mohammed, Andrew R. Green, Srinivasan Madhusudan, Emad A. Rakha

**Affiliations:** 1 Nottingham Breast Cancer Research Centre, Division of Cancer and Stem Cells, School of Medicine, The University of Nottingham, Nottingham, UK; 2 Department of Pathology, College of Dentistry, Al Mustansiriya University, Baghdad, Iraq; 3 Department of Pathology, Faculty of Medicine, Menoufia University, Menoufia, Egypt; 4 Department of Oncology, Nottingham University Hospitals, Nottingham, UK

**Keywords:** Flap Endonuclease 1 (FEN1), Ductal Carcinoma in situ, Prognosis, Breast Cancer

## Abstract

**Background:**

Impaired DNA repair mechanism is one of the cancer hallmarks. Flap Endonuclease 1 (FEN1) is essential for genomic integrity. FEN1 has key roles during base excision repair (BER) and replication. We hypothesised a role for FEN1 in breast cancer pathogenesis. This study aims to assess the role of FEN1 in breast ductal carcinoma in situ (DCIS).

**Methods:**

Expression of FEN1 protein was evaluated in a large (*n* = 1015) well-characterised cohort of DCIS, comprising pure (*n* = 776) and mixed (DCIS coexists with invasive breast cancer (IBC); *n* = 239) using immunohistochemistry (IHC).

**Results:**

FEN1 high expression in DCIS was associated with aggressive and high-risk features including higher nuclear grade, larger tumour size, comedo type necrosis, hormonal receptors negativity, higher proliferation index and triple-negative phenotype. DCIS coexisting with invasive BC showed higher FEN1 nuclear expression compared to normal breast tissue and pure DCIS but revealed significantly lower expression when compared to the invasive component. However, FEN1 protein expression in DCIS was not an independent predictor of local recurrence-free interval.

**Conclusion:**

High FEN1 expression is linked to features of aggressive tumour behaviour and may play a role in the direct progression of DCIS to invasive disease. Further studies are warranted to evaluate its mechanistic roles in DCIS progression and prognosis.

**Supplementary Information:**

The online version contains supplementary material available at 10.1007/s10549-021-06271-y.

## Introduction

Factors that affect DNA integrity and genome stability play a significant role in carcinogenesis [1, 2]. Genotoxic insults, which drive DNA damage, are a hallmark of cancer initiation and progression. These are induced by exogenous sources such as radiation, chemicals and environmental conditions, or intrinsic factors such as age-induced genetic changes and genetic predisposition [3–11] that lead to impaired DNA repair mechanisms [12, 13]. There are several mechanisms for DNA damage that include DNA double-strand breaks (DSBs), intra- and inter-strand DNA crosslinks, protein DNA adducts, methylated, mismatched and oxidised bases [13, 14]. DNA damage repair (DDR) is a complex mechanism, depends on the interaction between various pathways to repair damaged DNA [15].

Flap endonuclease 1 (FEN1) is recognised as a key enzyme that has a critical role in multiple DNA metabolic pathways involved in DNA replication, repair and apoptosis [16]. FEN1 has an essential role in cancer evolution and progression [17–19]. FEN1 belongs to the Rad2 structure-specific nuclease family, and it participates in Okazaki fragment maturation and recombination. FEN1 is recognised as a 5′ exonuclease (EXO activity) and gap endonuclease dependent (GEN activity) [26]. Therefore, FEN1 plays a vital role in maintaining genomic stability [20]. However, FEN1 is a pleiotropic protein with various functions. Three key interplaying mechanisms can regulate FEN1 functions including (I) Construction of complex with diverse proteins partner [21]. (II) Sub-cellular compartmentalisation: during DNA damage, FEN1 is localised in the nucleus [22], and superior localisation of FEN1 in the nucleolus may preserve the stability of the organisational structure of duplicated ribosomal DNA [23]. Moreover, the localisation of FEN1 in the mitochondrion has a vital function in repairing and replicating mitochondrial DNA (mtDNA) [24]. (III) Post-translational modifications: the ability of FEN1 to phosphorylate, methylate and acetylate proteins could be beneficial for the adjustment of the activities of the nuclease, protein partner and/or subcellular compartmentalisation [25, 26].

FEN1 mutations play a role in some autoimmune diseases, chronic inflammatory conditions and cancer predisposition. This suggests that mutator phenotype may initiate and develop cancer, while chronic inflammation promotes cancer progression [18]. Several studies showed that FEN1 is expressed intensively in proliferating cells with high DNA replication levels such as testes, bone marrow and thymus tissue [27–30]. In addition, FEN1 is upregulated in prostate cancer [19, 31], pancreatic cancer [32], gastric cancer [33], neuroblastoma [34] and lung cancer [35]. Some studies showed that FEN1 is upregulated in invasive breast cancer (IBC) compared to normal breast tissue. The dysregulation of FEN1 protein in breast and ovarian cancer is correlated with aggressive behaviour and worse outcome [16, 36].

Due to the controversy of FEN1 roles in cancer progression and behaviour and lack of studies describing its role in breast ductal carcinoma in situ (DCIS), we have hypothesised that FEN1 expression in DCIS plays a role in the disease progression. This study aims to assess the expression of FEN1 in a large cohort of pure DCIS and DCIS coexist with IBC using immunohistochemistry (IHC) and to determine its association with the various clinicopathological parameters and disease outcome.

## Material and methods

### Study cohort

This retrospective study was based on a large well-characterised cohort (*n* = 1015) diagnosed at the Breast Cancer Institute, Nottingham City Hospital, UK [37]. The study series comprised a primary pure DCIS (*n* = 776), and a cohort of DCIS coexists with IBC (*n* = 239). In addition, the adjacent apparently normal terminal ducto-lobular units (TDLUs) were assessed, whenever present (*n* = 65), among the included cases. Clinicopathological data of the pure DCIS cohort including age at diagnosis, disease presentation (screening or symptomatic), nuclear grade, presence of comedo necrosis, tumour size, type of surgery and postoperative radiotherapy were collected. Molecular classification of breast cancer based on the expression of oestrogen receptor (ER), progesterone receptor (PR), Her2 status and proliferation index Ki-67 index was performed as previously described [37]. ER and PR positivity were defined when more than or equal to 1% of the tumour cell nuclei showed positivity [38]. Her2 was assessed using the Herceptin test method, where IHC scoring of 0 or 1 was considered as negative, 2 + considered as equivocal and 3 + considered as positive [39]. Ki-67 proliferation index was defined as high if > 14% of malignant epithelial cells showed nuclear expression [40]. Local recurrence-free interval (LRFI) (in months) was estimated from the date of primary DCIS surgical treatment to the time of development of ipsilateral recurrence event as DCIS or IBC. Cases with positive tumour margin for patients who underwent re-excision in the first six months after breast-conserving surgery (BCS) and cases with contralateral breast event were censored. Pure DCIS median follow-up was 112 months (range 6–336). Out of the 1015 patients, only 95 (representing 9% of the whole cohort) in the primary DCIS series developed a local recurrence either in situ (34 cases; 36%) or IBC recurrence (61 cases; 64%). Supplementary Table S1 summarises the main demographic and clinicopathological parameters of the pure DCIS cohort.

### Analysis of *FEN1* mRNA in IBC

Due to the limited transcriptomic DCIS data, the Molecular Taxonomy of Breast Cancer International Consortium (METABRIC) (n = 1980) was used to validate the clinical implication and prognostic significance of *FEN1* in BC [41]. Moreover, analysis using the Breast Cancer Gene Expression Miner v4.1 (bc-GenExMiner v4.1) database was performed to evaluate the prognostic role of *FEN1* in IBC.

### FEN1 protein expression and immunohistochemistry

**Fig. 1:**
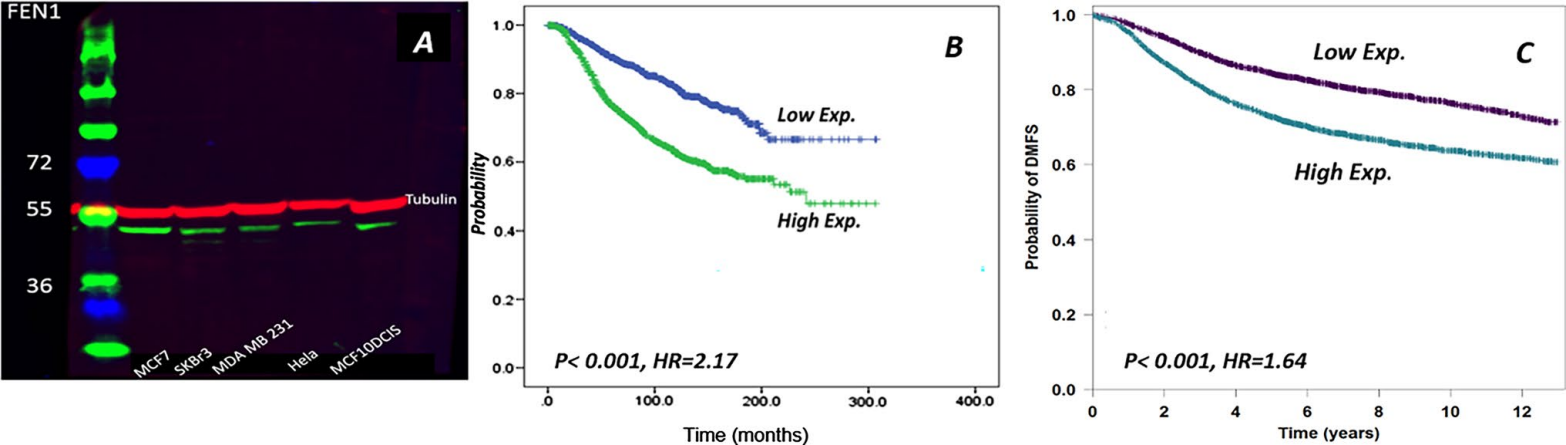
**a** FEN1 antibody validation, western blot showing single band (green band) of predicted size around 42.6 KDa in 5 cell lysates (MCF7, SKBr3, 231, Hela and MCF10DCIS). Tubulin used as a standard control shows a single band (red band) at 50 KDa. **b** Kaplan–Meier curve showing a high level of FEN1 mRNA expression in tumour breast epithelial cells associated with shorter breast cancer-specific survival 0f IBC within METABRIC cohort, **c** Kaplan–Meier curve showing high level of FEN1 was significantly associated with increased probability of distant metastasis and shorter overall survival in Breast Cancer Gene Expression Miner v4.2 (bc-GenExMiner v4.2)

Prior to IHC and to validate the antibody specificity, FEN1 antibody (Sigma; rabbit polyclonal, product number HPA00784, Lot number Ro7492) was validated using Western blot (WB) on a panel of human breast cell lysates: MCF7, SKBr3, MBA-MD-231 and MCF10DCIS that were obtained from the American Type Culture Collection; Rockville, MD, USA. FEN1 was used at a concentration of 1:1000 and showed a single specific band at the predicted size of approximately 43 kDa. Anti-tubulin antibody was used as a housekeeping marker (Abcam ab56676, Concentration 1:5000) which showed a single band at the expected molecular weight (55 kDa) (Fig. 1a).

IHC of FEN1 (dilution of 1:50) was performed on 4 µm tissue microarray (TMA) sections [37] using the Novocastra Novolink polymer detection system (Leica, Newcastle, UK) following the manufacturer’s guidelines. In addition, full-face tissue sections from 10 randomly selected cases were prepared to assess the heterogeneity of FEN1 protein expression prior to score the TMA sections. Tonsil was included as a positive control, whereas a negative control was achieved by omitting the primary antibody.

### FEN1 expression scoring

Semi-quantitative histochemical score (H-score) was used to assess FEN1 nuclear and cytoplasmic expression, including the intensity (negative, weak, moderate and strong expression as 0, 1, 2 and 3, respectively) multiplied by the percentage of stained tumour cells. The score was expressed in a range of 0–300 [42]. All cores that have less than 15% tumour or been folded or lost in processing were excluded. Dichotomisation of nuclear FEN1 staining into high (H-score > 70) and low (H-score ≤ 70), and cytoplasmic staining into high (H-score > 55) and low (H-score ≤ 50) was performed. Cut-off points were determined using X-tile (X- tile Bioinformatics software, University of Yale, version 3.6.1) [43]. Scoring was performed blind to clinicopathological data and patient outcome. Thirty percent of cases were double scored by another trained observer and the discrepant cases were reviewed, and a final score was agreed.

### Statistical analysis

SPSS software version 24 (Chicago. IL. USA) was used for statistical analysis. Based on the data distribution (parametric or non-parametric), appropriate statistical tests were carried out. Association between *FEN1* mRNA level and the clinicopathological parameters in the METABRIC database was performed using Chi-square test. The correlation between FEN1 protein expressions with the clinicopathological parameters was carried out by using Chi-square, Mann–Whitney and Kruskal–Wallis tests. To compare between FEN1 expression in apparently normal TDLU and DCIS, Wilcoxon-signed test was used. To compare between FEN1 expression in pure DCIS and the DCIS component in mixed cases, independent samples T-test were performed. Paired samples T-test and Wilcoxon Signed Ranks Test were performed to compare between FEN1 expression in mixed DCIS component and invasive component in the mixed cohort. Outcome analysis was carried out using log rank test and Kaplan–Meier. A P value of less than 0.05 was considered significant.

## Results

### *FEN1* mRNA expression in METABRIC data

High *FEN1* mRNA expression was observed in 50% of cases. High *FEN1* mRNA level was associated with younger patient age (*p* = 0.037), large tumour size (*p* < 0.001), high nuclear grade (*p* < 0.001), positive lymph node involvement (*p* < 0.001), hormonal receptor negativity (*p* < 0.001), positive HER2 status (*p* < 0.001) and basal-like subgroup (*p* < 0.001) (Supplementary Table S2). In addition, a high level of *FEN1* mRNA was predictive of short breast cancer-specific survival (BCSS) (*p* < 0.001, HR = 2.170, 95% CI = 1.355–2.031). Moreover, Breast Cancer Gene Expression Miner v4.2 (bc-GenExMiner v4.2) data demonstrate that high FEN1 was significantly associated with increased probability of distant metastasis and shorter overall survival (*p* < 0.001, HR = 1.64, 95% CI = 1.50–1.81) (Fig. 1b, c respectively).

### FEN1 protein expression

Full-face tissue sections revealed a homogenous staining pattern indicating the suitability of TMA for evaluation of FEN1 expression.

**Fig. 2:**
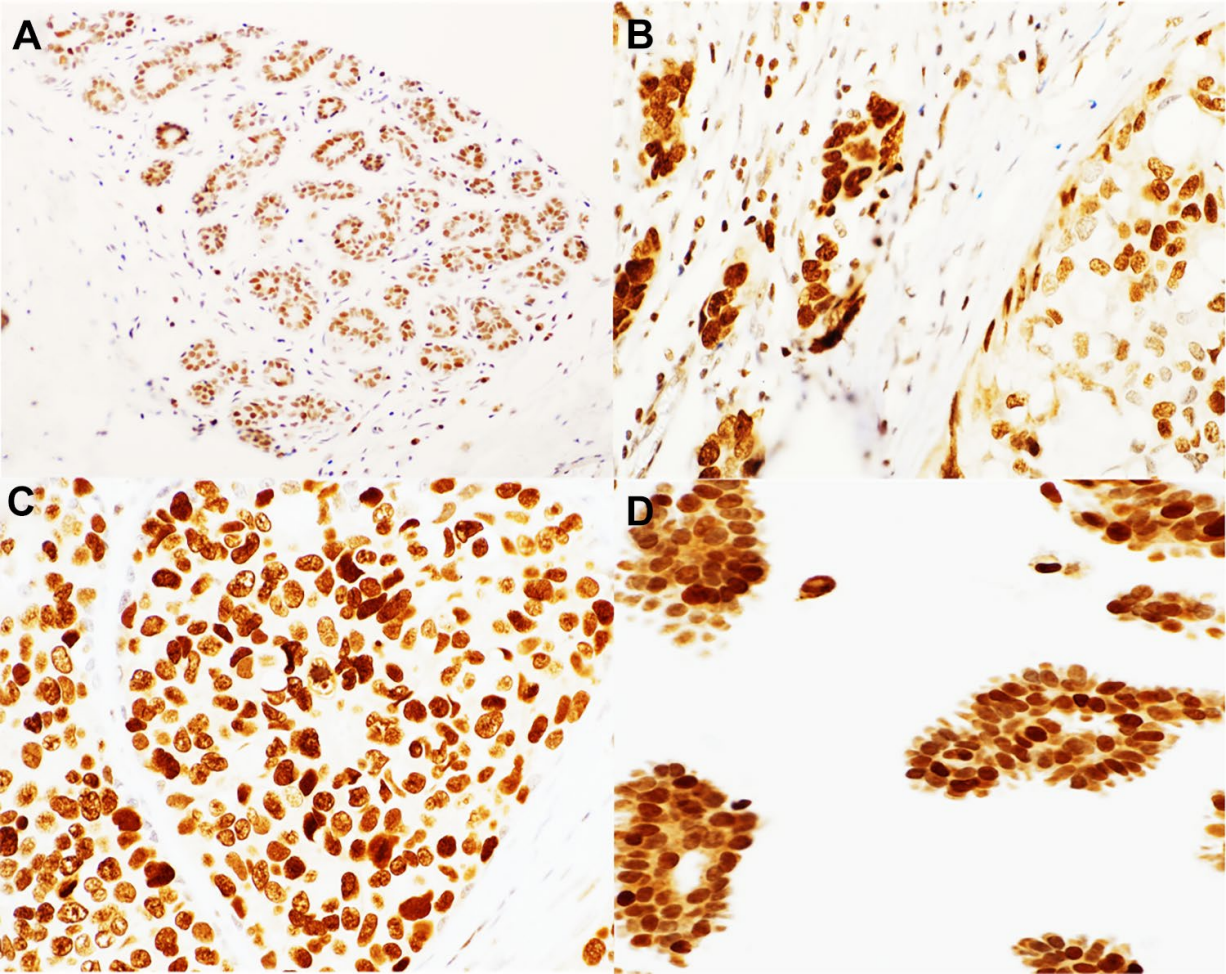
FEN1 protein expression in **a** Normal ducto-lobular units in human breast shows the weak expression of FEN1 and arrangement of the ducto-lobular units (X10). **b** Expression of FEN1 in a mixed DCIS/IBC case showing strong staining of FEN1 in IBC component than DCIS component (X40). **c** Strong nuclear expression of FEN1 in pure DCIS cancer cells (X40). **d** FEN1 expression in nuclear and Cytoplasmic cancer cells (X40)

A total of 437 pure DCIS cases were suitable for scoring and evaluation. FEN1 nuclear median H-score was 40 (range 0–120), 70 (range 0–230), 70 (range 0–200) and 85 (range 0–220) in TDLU, pure DCIS, DCIS component and the invasive component of the mixed cohort, respectively. FEN1 cytoplasmic median H-score was 10 (range 0–80), 50 (range 0–120), 70 (range 0–120) and 70 (range 0–100) in TDLU, pure DCIS cohort, DCIS component of the mixed cohort and IBC component, respectively (Fig. 2a–d).

High nuclear FEN1 expression was detected in 187 (43%) of pure DCIS cases. In the mixed cohort, FEN1 expression was higher in the invasive component compared to the DCIS component: 116 (62%) and 85 (46%) cases, respectively.

High cytoplasmic FEN1 protein expression was observed in 240 (55%) cases in pure DCIS. Within the mixed cohort, high cytoplasmic FEN1 expression was seen in 100 (54%) in the DCIS component and 91 (49%) of the invasive component.

### The correlation between FEN1 protein expression and clinicopathological parameters

High nuclear FEN1 expression was associated with aggressive clinicopathological variables in the pure DCIS cohort including larger tumour size (*p* = 0.008), high nuclear grade (*p* < 0.001), comedo type of necrosis (*p* < 0.001), negative hormonal status (*p* < 0.001), higher proliferation index (*p* < 0.001) and triple-negative tumours (*p* < 0.001) (Table 1). Similar results were shown when the analysis was carried out using the continuous data (Supplementary Table S3). High FEN1 cytoplasmic protein expression was significantly associated with negative hormone receptor status (*p* < 0.001), positive HER2 (*p* = 0.009), high proliferative index (Ki-67) (*p* = 0.042) and triple-negative subtype (*p* = 0.001) (Table 2) (Supplementary Table S4).

**Table 1:** Correlation between nuclear FEN1 protein expression and clinicopathological parameters in pure DCIS cohort using categorical values

Parameters	Low Exp. No. (%)	High Exp. No. (%)	Total No. (%)	(χ^2^) p-value
Age (years)				
≤ 50	65 (61.0)	41 (39.0)	106 (24.0)	0.967
> 50	185 (56.0)	146 (44.0)	331 (76.0)	0.325
Size				
≤ 20 mm	124 (64.0)	69 (36.0)	193 (44.0)	6.960
> 20 mm	125 (52.0)	117 (48.0)	242 (56.0)	**0.008**
DCIS presentation				
Screening	120 (57.0)	93 (44.0)	213 (49.0)	0.129
Symptomatic	130 (58.0)	94 (42.0)	224 (51.0)	0.750
Nuclear grade				
Low	41 (77.0)	12 (23.0)	53 (12.0)	**15.960**
Moderate	74 (64.0)	42 (36.0)	116 (27.0)	** < 0.001**
High	135 (50.0)	133 (50.0)	268 (61.0)	
Comedo necrosis				
No	108 (71.0)	45 (30.0)	153 (35.0)	**17.217**
Yes	142 (50.0)	142 (50.0)	284 (65.0)	** < 0.001**
Oestrogen receptor				
Negative	38 (36.0)	67 (64.0)	105 (27.0)	**31.108**
Positive	193 (67.0)	93 (33.0)	286 (73.0)	** < 0.001**
Progesterone receptor				
Negative	71 (44.0)	92 (56.0)	163 (41.0)	**24.882**
Positive	160 (69.0)	73 (31.0)	233 (59.0)	** < 0.001**
HER2 Status				
Negative	179 (61.0)	115 (39.0)	294 (76.0)	3.414
Positive	46 (50.0)	46 (50.0)	92 (24.0)	0.065
Proliferation index(Ki-67)				
Low (< 14%)	183 (67.0)	89 (33.0)	272 (76.0)	**45.394**
High (≥ 14%)	22 (26.0)	63 (74.0)	85 (24.0)	** < 0.001**
Molecular classes				
Luminal A	119 (72.0)	46 (28.0)	165 (50.0)	
Luminal B	38 (56.0)	30 (44.0)	68 (21.0)	**31.307**
HER2 enriched	16 (40.0)	24 (60.0)	40 (12.0)	** < 0.001**
Triple negative	20 (35.0)	37 (65.0)	57 (17.0)	

Significant *p* values are in bold, No: Number, X^2^: Chi square

*FEN1* Flap endonuclease 1, *DCIS* Ductal Carcinoma in Situ, *HER2* Human epidermal growth factor receptor2

**Table 2:** Correlation between cytoplasmic FEN1 protein expression in DCIS with clinicopathological parameters in pure DCIS cohort using categorical values

Parameters	Low Exp. No. (%)	High Exp. No. (%)	Total No. (%)	(χ^2^) *p*-value
Age (years)				
≤ 50	51 (48.0)	55 (52.0)	106 (24.0)	0.520
> 50	146 (44.0)	185 (56.0)	331 (76.0)	0.471
Size**				
≤ 20 mm	83 (43.0)	110 (57.0)	193 (44.0)	0.729
> 20 mm	114 (47.0)	128 (53.0)	242 (56.0)	0.393
DCIS presentation				
Screening	97 (46.0)	116 (54.0)	213 (49.0)	0.035
Symptomatic	100 (45.0)	124 (55.0)	224 (51.0)	0.851
Nuclear grade				
Low	30 (57.0)	23 (43.0)	53 (12.0)	3.560
Moderate	53 (53.0)	63 (54.0)	116 (27.0)	0.169
High	114 (43.0)	154(57.0)	268 (61.0)	
Comedo Necrosis				
No	78 (51.0)	75 (49.0)	153 (35.0)	3.310
Yes	119 (42.0)	165(58.0)	284 (65.0)	0.069
Oestrogen receptor				
Negative	31 (30.0)	74 (70.0)	105 (27.0)	**17.214**
Positive	125 (53.0)	134 (47.0)	286 (73.0)	** < 0.001**
Progesterone receptor				
Negative	55 (34.0)	108 (66.0)	163 (41.0)	**16.649**
Positive	127 (54.0)	106(46.0)	233 (59.0)	** < 0.001**
HER2 Status				
Negative	137 (47.0)	157 (53.0)	294 (76.0)	0.275
Positive	40 (44.0)	52 (56.0)	92 (24.0)	0.600
Proliferation index (Ki-67)				
Low (< 14%)	140 (51.0)	132 (49.0)	272 (76.0)	**4.147**
High (≥ 14%)	33 (39.0)	52 (61.0)	85 (24.0)	**0.042**
Molecular classes				
Luminal A	93 (56.0)	72 (44.0)	165 (50.0)	
Luminal B	34 (50.0)	34 (50.0)	68 (21.0)	**17.227**
HER2 enriched	15 (38.0)	25 (62.0)	40 (12.0)	**0.001**
Triple negative	15 (26.0)	42 (74.0)	57 (17.0)	

Significant *p* values are in bold, No Number, X^2^ Chi square

*FEN1* Flap endonuclease 1, *DCIS* Ductal Carcinoma in Situ, *HER2* Human epidermal growth factor receptor 2

Combined FEN1 nuclear/cytoplasmic protein expression was assessed in the pure DCIS cohort, where 142 cases (33%) showed high nuclear/high cytoplasmic (H.N/H.C), 152 cases (35%) showed low nuclear/low cytoplasmic (L.N/L.C), 98 cases (22%) showed low nuclear/high cytoplasmic L.N/H.C) and 45 cases (10%) showed high nuclear/low cytoplasmic (H.N/L.C) FEN1 expression**.** Cases with H.N/ H.C FEN1 expression were more likely expressed in DCIS with an aggressive behaviour: larger tumour size (*p* = 0.012), high nuclear grade (*p* = 0.003), comedo necrosis (*p* < 0.001), negative ER (*p* < 0.001), negative PR (*p* < 0.001), higher proliferation index (*p* < 0.001) and triple-negative subtype (*p* < 0.001) (Table 3).

**Table 3:** The correlation between Nuclear/Cytoplasmic (clustering) FEN1 protein expression in pure DCIS cohort with clinicopathological parameters

Parameters	H.N/ H.C No. (%)	H.N/ L.C No. (%)	L.N/ L.C No. (%)	L.N/ H.C No. (%)	Total No. (%)	χ2 *P* value
Age (years)						
≤ 50	33 (31.0)	43 (41.0)	22 (21.0)	8 (8.0)	106 (24.0)	2.628
> 50	109(33.0)	109(33.0)	76 (23.0)	37 (11.0)	331 (76.0)	0.453
DCIS Size						
≤ 20 mm	55 (29.0)	69 (36.0)	55 (29.0)	14 (7.0)	193 (44.0)	**10.888**
> 20 mm	86 (36.0)	83 (34.0)	42 (17.0)	31 (13.0)	242 (56.0)	**0.012**
DCIS Presentation						
Screening	69 (32.0)	73 (34.0)	47 (22.0)	24 (11.0)	213 (49.0)	0.436
Symptomatic	73 (33.0)	79 (35.0)	51 (23.0)	21 (9.0)	224 (51.0)	0.933
Nuclear Grade						
Low	10 (19.0)	28 (53.0)	13 (25.0)	2 (4.0)	53 (12.0)	**20.247**
Moderate	29 (25.0)	40 (35.0)	34 (29.0)	13 (11.0)	116 (27.0)	**0.003**
High	103(38.0)	84 (31.0)	51 (19.0)	30 (11.0)	268 (61.0)	
Comedo Necrosis						
No	32 (21.0)	65 (45.0)	43 (28.0)	13 (9.0)	153 (35.0)	17.855
Yes	110(39.0)	87 (31.0)	55 (19.0)	32 (11.0)	284 (65.0)	** < 0.001**
Oestrogen receptor						
Negative	54 (51.0)	18 (17.0)	20 (19.0)	13 (12.0)	105 (27.0)	**36.346**
Positive	66 (23.0)	125(44.0)	68 (24.0)	27 (9.0)	286 (73.0)	** < 0.001**
Progesterone receptor						
Negative	72 (44.0)	35 (22.0)	36 (22.0)	20 (12.0)	163 (41.0)	**24.842**
Positive	52 (22.0)	106(46.0)	45 (23.0)	21 (9.0)	233 (59.0)	** < 0.001**
HER2 Status						
Negative	85 (29.0)	107(36.0)	72 (25.0)	30 (10.0)	294 (76.0)	3.653
Positive	35 (38.0)	29 ( 32.0)	17 (19.0)	11 (12.0)	92 (24.0)	0.301
Proliferation index (Ki-67						
Low (≤ 14%)	66 (24.0)	117(43.0)	66 (24.0)	23 (9.0)	272 (76.0)	**45.779**
High (> 14%)	46 (54.0)	16 (19.0)	6 (7.0)	17 (20.0)	85 (24.0)	** < 0.001**
Molecular classes						
Luminal A	31 (19.0)	78 (47.0)	41 (25.0)	15 (9.0)	165 (50.0)	38.922
Luminal B	23 (34.0)	27 (40.0**)**	11 (16.0)	7 (10.0)	68 (21.0)	** < 0.001**
HER2 enriched	17 (43.0)	8 (20.0)	8 (20.0)	7 (18.0)	40 (12.0)	
Triple Negative	31 (54.0)	9 (16.0)	11 (19.0)	6 (11.0)	57 (17.0)	

Significant *p* values are in bold

*FEN1* Flap endonuclease 1, *DCIS* ductal carcinoma in situ, *HER2* Enriched; Human epidermal growth factor receptor 2. H.N/ H.C; High Nuclear/ Low Cytoplasmic expression. H.N/ L.C; High Nuclear/ Low Cytoplasmic expression. L.N/ L.C; Low Nuclear/ Low Cytoplasmic expression. L.N/ H.C; Low Nuclear/ High Cytoplasmic expression

No significant association was observed between FEN1 expression and the DCIS outcome in terms of ipsilateral recurrence.

FEN1 nuclear protein level of the adjacent TDLUs epithelial cells revealed the lowest level. The proportion of cases with high nuclear FEN1 expression in apparently normal TDLUs was lower than in DCIS (*p* = 0.024). IHC assessment exhibits a higher nuclear protein level of FEN1 in the DCIS component in a mixed cohort than the primary DCIS cohort (*p* = 0.032). Similarly, in the mixed DCIS/IBC cohort, the FEN1 nuclear protein level in the invasive component was higher than in the DCIS component (*p* < 0.001).

Within the DCIS mixed cohort, high nuclear FEN1 expression was observed in 85/185 cases (46%) in the DCIS component and 100/185 cases (54%) showed high cytoplasmic expression. High nuclear FEN1 expression in the DCIS component of the mixed cohort was associated with higher nuclear grade (*p* < 0.001), DCIS comedo necrosis (*p* < 0.001) and negative ER status (*p* = 0.002), while FEN1 cytoplasmic expression did not reveal any significant association in statistical analysis.

## Discussion

DCIS is a non-obligatory precursor of IBC [44]. Despite the massive similarity between IBC and DCIS at the molecular levels, the proposed similarity stemmed from the fact that cells that have progressed into invasive carcinomas were indeed originated from DCIS. Consequently, this could have a similar impact on the response of neoadjuvant and adjuvant therapy in both DCIS and IBC [45, 46].

Several studies demonstrated that FEN1 has a dual function. Alteration of FEN1 in cancer cells makes it a potential target for anticancer therapy [27, 47, 48]. Overexpression of FEN1 has been reported in previous studies such as in breast [36], prostate, testis, lung, brain and gastric tumours [49, 50]. Some studies found that FEN1 protein overexpression promotes cell growth, as reported by Kim et al. [28] and He et al. [51], and that the level of FEN1 was induced while DNA is replicating during cell proliferation. Consequently, this increased FEN1 level may have a role in the resistance to other DNA damage agents which eventually leads to an increased rate of cancer cell proliferation. Furthermore, FEN1 overexpression might be one of the main reasons for genome instability and impaired DNA replication in cancer cells [52, 53]. FEN1 overexpression has been proved to be associated with aggressive behaviour and poor survival in different tumours [36, 50].

The current study showed that high FEN1 expression is significantly associated with aggressive behaviour of DCIS. Similar to previous studies [54–56], the current study showed high expression of FEN1 is associated with ER and PR negativity. Abdel-Fatah et al. [36] reported that FEN1 could interact directly with ER and increase the interaction of ER-α with DNA-containing oestrogen response elements and impact the expression of oestrogen-responsive genes in cells. Lari and Kuerer [57] reported that DCIS patients with ER negative tumours were more likely to have a local recurrence than ER-positive patients. In previous studies, Schultz et al. [58] and Wang et al. [59] showed the association between the double function of FEN1 with the ER receptor in epithelial cells where FEN1 influences ER-α-mediated gene expression in epithelial cells in different ways depending on the presence or absence of 17β-Estradiole 2 (E2).

This study showed a positive correlation between high FEN1 protein levels with aggressive clinicopathological parameters including hormone status negativity, which can promote FEN1 as a good candidate biomarker for prognostication of DCIS according to their hormonal status. It has been reported the dual function of FEN1, based on its expression, was found to be inducible to DNA synthesis during cell proliferation and is down-regulated during cell differentiation [27]. Depletion and/or inhibition of FEN1 activity elevate endogenous DNA damage sensitivity to alkylating insults [13, 47]. However, long exposure to DNA alkylating insult may ultimately generate adapted cancer cells to these agents [60]. In tumour cells, down regulation of FEN1 protein could enhance DNA-damaged inducing agent’s toxicity leading to both DNA replication and repair failure. Because of the high rate of replication, cancerous cells accumulate and tend to promote innate DNA damage compared to adjacent normal cells. Moreover, post-phosphorylation activates the P53 pathway which is the most common pathway of apoptosis (*Tp53*-dependent apoptosis) and acts as a transcription process to stimulate the expression of genes involved in apoptosis. Besides, cell proliferation was suppressed by the FEN1 inhibitor and stimulates DNA damage; consequently, the accumulation of unrepaired double-strand breaks elevates the proportion of G1 phase and decreases the proportion of S and G2/M phase in the cell cycle. This could mean FEN1 protein is essential for the cells to enter S phase, otherwise cells will be arrested [51]. Cytotoxicity sensitivity of anticancer drugs may be increased by FEN1 depletion and/or inhibition. Inducing the cytotoxicity could promote impaired DNA repair and replication [47, 48].

We also investigated the nuclear/cytoplasmic clusters which revealed that DCIS who had high nuclear/high cytoplasmic clusters were most likely associated with aggressive DCIS behaviour. This observation supports our initial results in nuclear protein expression and cytoplasmic protein expression revealing an association between high protein levels and aggressive tumour behaviour. Our data of FEN1 protein expression in patients who had primary DCIS did not show any significant association with patient’s outcome. This lack of association could be due to the limited number of patients who had an ipsilateral recurrence in the study cohort.

As previously discussed, depletion and/or inhibition of FEN1 activity showed more impact risk on tumour cells than in adjacent normal cells, which revealed the poor outcome for patients who had DCIS. Although a high protein level of FEN1 was associated with clinicopathological parameters characteristics of poor prognosis, multivariate analysis did not show an independent prognostic value of FEN1 expression in DCIS patients underwent BCS treatment. This may be due to the limited number of patients who developed ipsilateral recurrence. We recommend further functional and mechanistic studies to clarify the specific roles of FEN1 in DCIS.

This study has some limitations. All the samples in this study were obtained from patients diagnosed in one centre from the city hospital in Nottingham UK, and for more verification, samples can be retrieved and used from multiple centres. Moreover, the study was performed on a cohort of patients that were not treated with endocrine therapy. In addition, the study was performed on TMA sections. Although all cases were reviewed histologically before construction and multiple cores with heterogeneous grades and morphological patterns were used for the cases, they might still underestimate the heterogeneity of the tumour roles.

## Conclusion

Our data present evidence that high FEN1 protein level is associated with aggressive behaviour in the DCIS and could be an indicator for progression from DCIS into IBC. Our speculation is that FEN1 may have different roles in the nucleus and cytoplasm.

## Supplementary Information

Below is the link to the electronic supplementary material.

Supplementary file1 (DOCX 38 kb)

## Data Availability

The authors confirm the data that have been used in this work are available at a reasonable request.

## References

[1] Balmain A, Gray J, Ponder B (2003). The genetics and genomics of cancer. Nat Genet.

[2] Nathanson KL, Wooster R, Weber BL (2001). Breast cancer genetics: what we know and what we need. Nat Med.

[3] Marteijn JA, Lans H, Vermeulen W, Hoeijmakers JH (2014). Understanding nucleotide excision repair and its roles in cancer and ageing. Nat Rev Mol Cell Biol.

[4] Ciccia A, Elledge SJ (2010). The DNA damage response: making it safe to play with knives. Mol Cell.

[5] Rudel RA, Attfield KR, Schifano JN, Brody JG (2007). Chemicals causing mammary gland tumors in animals signal new directions for epidemiology, chemicals testing, and risk assessment for breast cancer prevention. Cancer.

[6] Garcia E, Hurley S, Nelson DO, Hertz A, Reynolds P (2015). Hazardous air pollutants and breast cancer risk in California teachers: a cohort study. Environmental health : a global access science source.

[7] Grant WB (2014). Solar ultraviolet irradiance and cancer incidence and mortality. Adv Exp Med Biol.

[8] Singh JC, Lichtman SM (2015). Effect of age on drug metabolism in women with breast cancer. Expert Opin Drug Metab Toxicol.

[9] Liu Y, Nguyen N, Colditz GA (2015). Links between alcohol consumption and breast cancer: a look at the evidence. Womens Health (Lond Engl).

[10] Terry PD, Rohan TE (2002). Cigarette smoking and the risk of breast cancer in women: a review of the literature. Cancer epidemiology, biomarkers & prevention : a publication of the American Association for Cancer Research, cosponsored by the American Society of Preventive Oncology.

[11] Faraglia B, Chen SY, Gammon MD, Zhang Y, Teitelbaum SL, Neugut AI, Ahsan H, Garbowski GC, Hibshoosh H, Lin D, Kadlubar FF, Santella RM (2003). Evaluation of 4-aminobiphenyl-DNA adducts in human breast cancer: the influence of tobacco smoke. Carcinogenesis.

[12] Broustas CG, Lieberman HB (2014). DNA damage response genes and the development of cancer metastasis. Radiat Res.

[13] Ali R, Rakha EA, Madhusudan S, Bryant HE (2017). DNA damage repair in breast cancer and its therapeutic implications. Pathology.

[14] White AJ, Chen J, McCullough LE, Xu X, Cho YH, Teitelbaum SL, Neugut AI, Terry MB, Hibshoosh H, Santella RM, Gammon MD (2015). Polycyclic aromatic hydrocarbon (PAH)-DNA adducts and breast cancer: modification by gene promoter methylation in a population-based study. Cancer Causes Control.

[15] Horton JK, Baker A, Berg BJ, Sobol RW, Wilson SH (2002). Involvement of DNA polymerase beta in protection against the cytotoxicity of oxidative DNA damage. DNA Repair.

[16] Singh P, Yang M, Dai H, Yu D, Huang Q, Tan W, Kernstine KH, Lin D, Shen B (2008). Overexpression and hypomethylation of flap endonuclease 1 gene in breast and other cancers. Molecular cancer research : MCR.

[17] Kucherlapati M, Yang K, Kuraguchi M, Zhao J, Lia M, Heyer J, Kane MF, Fan K, Russell R, Brown AM, Kneitz B, Edelmann W, Kolodner RD, Lipkin M, Kucherlapati R (2002). Haploinsufficiency of Flap endonuclease (Fen1) leads to rapid tumor progression. Proc Natl Acad Sci USA.

[18] Zheng L, Dai H, Zhou M, Li M, Singh P, Qiu J, Tsark W, Huang Q, Kernstine K, Zhang X, Lin D, Shen B (2007). Fen1 mutations result in autoimmunity, chronic inflammation and cancers. Nat Med.

[19] Lam JS, Seligson DB, Yu H, Li A, Eeva M, Pantuck AJ, Zeng G, Horvath S, Belldegrun AS (2006). Flap endonuclease 1 is overexpressed in prostate cancer and is associated with a high Gleason score. BJU Int.

[20] Singh P, Zheng L, Chavez V, Qiu J, Shen B (2007). Concerted action of exonuclease and Gap-dependent endonuclease activities of FEN-1 contributes to the resolution of triplet repeat sequences (CTG)n- and (GAA)n-derived secondary structures formed during maturation of Okazaki fragments. J Biol Chem.

[21] Kathera C, Zhang J, Janardhan A, Sun H, Ali W, Zhou X, He L, Guo Z (2017). Interacting partners of FEN1 and its role in the development of anticancer therapeutics. Oncotarget.

[22] Qiu J, Li X, Frank G, Shen B (2001). Cell cycle-dependent and DNA damage-inducible nuclear localization of FEN-1 nuclease is consistent with its dual functions in DNA replication and repair. J Biol Chem.

[23] Guo Z, Qian L, Liu R, Dai H, Zhou M, Zheng L, Shen B (2008). Nucleolar localization and dynamic roles of flap endonuclease 1 in ribosomal DNA replication and damage repair. Mol Cell Biol.

[24] Liu P, Qian L, Sung JS, de Souza-Pinto NC, Zheng L, Bogenhagen DF, Bohr VA, Wilson DM, Shen B, Demple B (2008). Removal of oxidative DNA damage via FEN1-dependent long-patch base excision repair in human cell mitochondria. Mol Cell Biol.

[25] Hasan S, Stucki M, Hassa PO, Imhof R, Gehrig P, Hunziker P, Hubscher U, Hottiger MO (2001). Regulation of human flap endonuclease-1 activity by acetylation through the transcriptional coactivator p300. Mol Cell.

[26] Guo Z, Zheng L, Xu H, Dai H, Zhou M, Pascua MR, Chen QM, Shen B (2010). Methylation of FEN1 suppresses nearby phosphorylation and facilitates PCNA binding. Nat Chem Biol.

[27] Kim IS (1998). Down-regulation of human FEN-1 gene expression during differentiation of promyelocytic leukemia cells. Exp Mol Med.

[28] Kim IS, Lee MY, Lee IH, Shin SL, Lee SY (2000). Gene expression of flap endonuclease-1 during cell proliferation and differentiation. Biochem Biophys Acta.

[29] Warbrick E, Coates PJ, Hall PA (1998). Fen1 expression: a novel marker for cell proliferation. J Pathol.

[30] Otto CJ, Almqvist E, Hayden MR, Andrew SE (2001). The "flap" endonuclease gene FEN1 is excluded as a candidate gene implicated in the CAG repeat expansion underlying Huntington disease. Clin Genet.

[31] LaTulippe E, Satagopan J, Smith A, Scher H, Scardino P, Reuter V, Gerald WL (2002). Comprehensive gene expression analysis of prostate cancer reveals distinct transcriptional programs associated with metastatic disease. Can Res.

[32] Iacobuzio-Donahue CA, Maitra A, Olsen M, Lowe AW, van Heek NT, Rosty C, Walter K, Sato N, Parker A, Ashfaq R, Jaffee E, Ryu B, Jones J, Eshleman JR, Yeo CJ, Cameron JL, Kern SE, Hruban RH, Brown PO, Goggins M (2003). Exploration of global gene expression patterns in pancreatic adenocarcinoma using cDNA microarrays. Am J Pathol.

[33] Kim JM, Sohn HY, Yoon SY, Oh JH, Yang JO, Kim JH, Song KS, Rho SM, Yoo HS, Kim YS, Kim JG, Kim NS (2005). Identification of gastric cancer-related genes using a cDNA microarray containing novel expressed sequence tags expressed in gastric cancer cells. Clinical cancer research : an official journal of the American Association for Cancer Research.

[34] Krause A, Combaret V, Iacono I, Lacroix B, Compagnon C, Bergeron C, Valsesia-Wittmann S, Leissner P, Mougin B, Puisieux A (2005). Genome-wide analysis of gene expression in neuroblastomas detected by mass screening. Cancer Lett.

[35] Sato M, Girard L, Sekine I, Sunaga N, Ramirez RD, Kamibayashi C, Minna JD (2003). Increased expression and no mutation of the Flap endonuclease (FEN1) gene in human lung cancer. Oncogene.

[36] Abdel-Fatah TM, Russell R, Albarakati N, Maloney DJ, Dorjsuren D, Rueda OM, Moseley P, Mohan V, Sun H, Abbotts R, Mukherjee A, Agarwal D, Illuzzi JL, Jadhav A, Simeonov A, Ball G, Chan S, Caldas C, Ellis IO, Wilson DM, Madhusudan S (2014). Genomic and protein expression analysis reveals flap endonuclease 1 (FEN1) as a key biomarker in breast and ovarian cancer. Mol Oncol.

[37] Miligy IM, Gorringe KL, Toss MS, Al-Kawaz AA, Simpson P, Diez-Rodriguez M, Nolan CC, Ellis IO, Green AR, Rakha EA (2018). Thioredoxin-interacting protein is an independent risk stratifier for breast ductal carcinoma in situ. Mod Pathol.

[38] Hammond MEH, Hayes DF, Dowsett M, Allred DC, Hagerty KL, Badve S, Fitzgibbons PL, Francis G, Goldstein NS, Hayes M, Hicks DG, Lester S, Love R, Mangu PB, McShane L, Miller K, Osborne CK, Paik S, Perlmutter J, Rhodes A, Sasano H, Schwartz JN, Sweep FCG, Taube S, Torlakovic EE, Valenstein P, Viale G, Visscher D, Wheeler T, Williams RB, Wittliff JL, Wolff AC (2010). American Society of Clinical Oncology/College Of American Pathologists guideline recommendations for immunohistochemical testing of estrogen and progesterone receptors in breast cancer. J Clin Oncol.

[39] Rakha EA, Pinder SE, Bartlett JMS, Ibrahim M, Starczynski J, Carder PJ, Provenzano E, Hanby A, Hales S, Lee AHS, Ellis IO, Committee NC (2015). Updated UK Recommendations for HER2 assessment in breast cancer. J Clin Pathol.

[40] Toss MS, Miligy IM, Gorringe KL, Aleskandarany MA, Alkawaz A, Mittal K, Aneja R, Ellis IO, Green AR, Rakha EA (2019). Collagen XI alpha-1 chain is an independent prognostic factor in breast ductal carcinoma in situ. Mod Pathol.

[41] Curtis C, Shah SP, Chin SF, Turashvili G, Rueda OM, Dunning MJ, Speed D, Lynch AG, Samarajiwa S, Yuan Y, Graf S, Ha G, Haffari G, Bashashati A, Russell R, McKinney S, Langerod A, Green A, Provenzano E, Wishart G, Pinder S, Watson P, Markowetz F, Murphy L, Ellis I, Purushotham A, Borresen-Dale AL, Brenton JD, Tavare S, Caldas C, Aparicio S (2012). The genomic and transcriptomic architecture of 2,000 breast tumours reveals novel subgroups. Nature.

[42] Chrysanthou E, Gorringe KL, Joseph C, Craze M, Nolan CC, Diez-Rodriguez M, Green AR, Rakha EA, Ellis IO, Mukherjee A (2017). Phenotypic characterisation of breast cancer: the role of CDC42. Breast Cancer Res Treat.

[43] Camp RL, Dolled-Filhart M, Rimm DL (2004). X-tile: a new bio-informatics tool for biomarker assessment and outcome-based cut-point optimization. Clinical Cancer Res.

[44] Waldman FM, DeVries S, Chew KL, Moore DH, Kerlikowske K, Ljung BM (2000). Chromosomal alterations in ductal carcinomas in situ and their in situ recurrences. J Natl Cancer Inst.

[45] Gorringe KL, Fox SB (2017). Ductal carcinoma in situ biology, biomarkers, and diagnosis. Front Oncol.

[46] O'Brien KM, Sun J, Sandler DP, DeRoo LA, Weinberg CR (2015). Risk factors for young-onset invasive and in situ breast cancer. Cancer causes & control : CCC.

[47] He L, Zhang Y, Sun H, Jiang F, Yang H, Wu H, Zhou T, Hu S, Kathera CS, Wang X, Chen H, Li H, Shen B, Zhu Y, Guo Z (2016). Targeting DNA flap endonuclease 1 to impede breast cancer progression. EBioMedicine.

[48] van Pel DM, Barrett IJ, Shimizu Y, Sajesh BV, Guppy BJ, Pfeifer T, McManus KJ, Hieter P (2013). An evolutionarily conserved synthetic lethal interaction network identifies FEN1 as a broad-spectrum target for anticancer therapeutic development. PLoS Genet.

[49] Zhang K, Keymeulen S, Nelson R, Tong TR, Yuan YC, Yun X, Liu Z, Lopez J, Raz DJ, Kim JY (2018). Overexpression of flap endonuclease 1 correlates with enhanced proliferation and poor prognosis of non-small-cell lung cancer. Am J Pathol.

[50] Wang K, Xie C, Chen D (2014). Flap endonuclease 1 is a promising candidate biomarker in gastric cancer and is involved in cell proliferation and apoptosis. Int J Mol Med.

[51] He L, Luo L, Zhu H, Yang H, Zhang Y, Wu H, Sun H, Jiang F, Kathera CS, Liu L, Zhuang Z, Chen H, Pan F, Hu Z, Zhang J, Guo Z (2017). FEN1 promotes tumor progression and confers cisplatin resistance in non-small-cell lung cancer. Mol Oncol.

[52] Becker JR, Gallo D, Leung W, Croissant T, Thu YM, Nguyen HD, Starr TK, Brown GW, Bielinsky AK (2018). Flap endonuclease overexpression drives genome instability and DNA damage hypersensitivity in a PCNA-dependent manner. Nucleic Acids Res.

[53] Moggs JG, Murphy TC, Lim FL, Moore DJ, Stuckey R, Antrobus K, Kimber I, Orphanides G (2005). Anti-proliferative effect of estrogen in breast cancer cells that re-express ERalpha is mediated by aberrant regulation of cell cycle genes. J Mol Endocrinol.

[54] Dobrescu A, Chang M, Kirtani V, Turi GK, Hennawy R, Hindenburg AA (2011). Study of estrogen receptor and progesterone receptor expression in breast ductal carcinoma in situ by immunohistochemical staining in er/pgr-negative invasive breast cancer. ISRN Oncology.

[55] Swain SM, Wilson JW, Mamounas EP, Bryant J, Wickerham DL, Fisher B, Paik S, Wolmark N (2004). Estrogen receptor status of primary breast cancer is predictive of estrogen receptor status of contralateral breast cancer. J Natl Cancer Inst.

[56] Arpino G, Weiss HL, Clark GM, Hilsenbeck SG, Osborne CK (2005). Hormone receptor status of a contralateral breast cancer is independent of the receptor status of the first primary in patients not receiving adjuvant tamoxifen. J Clin Oncol.

[57] Lari SA, Kuerer HM (2011). Biological markers in DCIS and risk of breast recurrence: a systematic review. J Cancer.

[58] Schultz-Norton JR, Walt KA, Ziegler YS, McLeod IX, Yates JR, Raetzman LT, Nardulli AM (2007). The deoxyribonucleic acid repair protein flap endonuclease-1 modulates estrogen-responsive gene expression. Molecular endocrinology (Baltimore, Md).

[59] Wang Y, Li S, Zhu L, Zou J, Jiang X, Chen M, Chen B (2019). Letrozole improves the sensitivity of breast cancer cells overexpressing aromatase to cisplatin via down-regulation of FEN1. Clinical & translational oncology : official publication of the Federation of Spanish Oncology Societies and of the National Cancer Institute of Mexico.

[60] Chen KH, Yakes FM, Srivastava DK, Singhal RK, Sobol RW, Horton JK, Van Houten B, Wilson SH (1998). Up-regulation of base excision repair correlates with enhanced protection against a DNA damaging agent in mouse cell lines. Nucleic Acids Res.

