# Supplementary material for: The prognostic significance of Flap Endonuclease 1 (FEN1) in breast ductal carcinoma in situ
Source: Breast Cancer Res Treat. 2021 Jun 12;188(1):53–63. doi: 10.1007/s10549-021-06271-y (PMC8233293; doi:10.1007/s10549-021-06271-y)
Supplement: Supplementary file 1 — Supplementary file1 (DOCX 38 kb) [file 10549_2021_6271_MOESM1_ESM.docx]

**Supplementary Tables and Figures**

**Supplementary Table S1**: Patient demographic and clinicopathological characters in pure DCIS series.

| **Clinicopathological parameter** | **Groups** | **Number of patients (%)** |
| --- | --- | --- |
| Age at diagnosis (years) | ≤50 | 232 (22) |
|  | >50 | 827 (78) |
| Method of Presentation | Screening | 606 (57) |
|  | Symptomatic | 453 (43) |
| DCIS Size (mm) | ≤20 | 542 (52) |
|  | >20 | 510 (48) |
| Nuclear Grade | Low | 143 (14) |
|  | Intermediate | 268 (25) |
|  | High | 644 (61) |
| Comedo Necrosis | Yes | 695 (66) |
|  | No | 364 (34) |
| Treatment group | Mastectomy | 542 (51) |
|  | Breast conserving surgery | 340 (32) |
|  | Breast conserving surgery +Radiotherapy | 176 (17) |
| Recurrence | Yes | 95 (9) |
|  | No | 964 (91) |
| Oestrogen Receptor status | Negative | 156 (22) |
|  | Positive | 545 (78) |
| Progesterone Receptor status | Negative | 260 (43) |
|  | Positive | 340 (57) |
| HER2 status | Negative | 517 (80) |
|  | Positive | 131 (20) |
| Proliferation index (Ki 67) | Low proliferation | 389 (77) |
|  | High proliferation | 118 (23) |
| Molecular classes | Luminal A | 248 (52) |
|  | Luminal B | 88 (19) |
|  | HER2 | 64 (14) |
|  | Triple negative | 73 (15) |

DCIS: Ductal carcinoma in situ, HER2; Human epidermal growth factor receptor

**Supplementary Table S 2:** Correlation between *FEN1* mRNA and clinicopathological parameters in the METABRIC cohort.

| *Parameters* | *Low Exp.*  *No. (%)* | *High Exp.*  *No. (%)* | *Total* | *χ^2^*  *P value* |
| --- | --- | --- | --- | --- |
| *Age (Years)*  *≤50*  *>50* | *192 (46.0)*  *773 (51.0)* | *228 (54.0)*  *729 (49.0)* | *420 (22.0)*  *1502 (78.0)* | ***4.341***  ***0.037*** |
| *Size*  *≤20mm*  *>20mm* | *469 (55.0)*  *505 (46.0)* | *379 (45.0)*  *587 (54.0)* | *848 (44.0)*  *1092 (56.0)* | ***15.677***  ***<0.001*** |
| *Nuclear Grade*  *Low*  *Moderate*  *High* | *144 (85.0)*  *492 (64.0)*  *292 (31.0)* | *25 (15.0)*  *272 (36.0)*  *656 (69.0)* | *169 (9.0)*  *764 (41.0)*  *948 (50.0)* | ***286.626***  ***<0.001*** |
| *Oestrogen Receptor*  *Negative*  *Positive* | *118 (25.0)*  *868 (58.0)* | *354 (75.0)*  *627 (42.0)* | *472 (24.0)*  *1495 (76.0)* | ***156.838***  ***<0.001*** |
| *Progesterone Receptor*  *Negative*  *Positive* | *352 (38.0)*  *634 (61.0)* | *584 (62.0)*  *397 (39.0)* | *936 (48.0)*  *1031 (52.0)* | ***111.972***  ***<0.001*** |
| *Her2 Status*  *Negative*  *Positive* | *915 (53.0)*  *71 (29.0)* | *807 (47.0)*  *174 (71.0)* | *1722 (88.0)*  *174 (12.0)* | ***50.063***  ***<0.001*** |
| *Molecular classes*  *Normal*  *Basal*  *Luminal A*  *Luminal B*  *HER2 enriched* | *162 (81.0*  *50 (15.0)*  *569 (80.0)*  *139 (29.0)*  *63 (27.0)* | *37 (19.0)*  *279 (85.0)*  *142 (20.0)*  *347 (71.0)*  *174 (73.0)* | *199 (10.0)*  *329 (17.0)*  *711 (36.0)*  *486 (25.0)*  *237 (12.0)* | ***635.355***  ***<0.001*** |

Significant *p* values are in **bold.** No: Number, X^2^: Chi square

FEN1; Flap endonuclease 1, DCIS; Ductal Carcinoma *in Situ.* METABRIC: Molecular Taxonomy of Breast Cancer International Consortium. HER2; Human epidermal growth factor receptor 2.

**Supplementary Table S 3:** Correlation between nuclear FEN1 protein expression in DCIS with clinicopathological parameters in pure DCIS cohort using continuous values.

| *Parameters* | *No. of cases* | *Mean Rank* | *p-value* |
| --- | --- | --- | --- |
| *Age*  *≤50*  *>50* | *106*  *331* | *203.75*  *223.89* | *0.152* |
| *DCIS Presentation*  *Screening*  *Symptomatic* | *213*  *224* | *227.23*  *211.17* | *0.183* |
| *Nuclear Grade*  *Low*  *Moderate*  *High* | *53*  *116*  *268* | *163.65*  *204.70*  *236.14* | ***<0.001*** |
| *Comedo Necrosis*  *No*  *Yes* | *153*  *284* | *185.75*  *236.92* | ***<0.001*** |
| *Oestrogen Receptor*  *Negative*  *Positive* | *105*  *286* | *245.43*  *177.85* | ***<0.001*** |
| *Progesterone Receptor*  *Negative*  *Positive* | *163*  *233* | *234.16*  *173.55* | ***<0.001*** |
| *HER2 Status*  *Negative*  *Positive* | *294*  *92* | *187.87*  *211.48* | *0.076* |
| *Proliferation index (Ki 67)*  *Low (<14%)*  *High (≥14%)* | *272*  *85* | *157.92*  *246.46* | ***<0.001*** |
| *Molecular classes*  *Luminal A*  *Luminal B*  *HER2 enriched*  *Triple Negative* | *165*  *68*  *40*  *57* | *136.12*  *180.22*  *194.71*  *212.50* | ***<0.001*** |

Significant *p* values are in **bold.** Mean rank operated by Mann Whitey test and Kruskal test. No: Number. FEN1; Flap endonuclease 1. DCIS; Ductal Carcinoma *in Situ*. HER2; Human epidermal growth factor receptor 2.

**Supplementary Table S 4:** Correlation between cytoplasmic FEN1 expression in DCIS with clinicopathological parameters in pure DCIS cohort using continuous values.

| *Parameters* | *No. of cases* | *Mean Rank* | *p-value* |
| --- | --- | --- | --- |
| *Age*  *≤50*  *>50* | *106*  *331* | *211.75*  *221.32* | *0.495* |
| *DCIS Presentation*  *Screening*  *Symptomatic* | *213*  *224* | *215.03*  *222.78* | *0.519* |
| *Nuclear Grade*  *Low*  *Moderate*  *High* | *53*  *116*  *268* | *190.10*  *218.47*  *224.94* | *0.182* |
| *Comedo Necrosis*  *No*  *Yes* | *153*  *284* | *205.30*  *226.38* | *0.094* |
| *Oestrogen Receptor*  *Negative*  *Positive* | *105*  *286* | *236.53*  *181.12* | ***<0.001*** |
| *Progesterone Receptor*  *Negative*  *Positive* | *163*  *233* | *228.16*  *177.75* | ***<0.001*** |
| *HER2 Status*  *Negative*  *Positive* | *294*  *92* | *191.42*  *200.15* | *0.511* |
| *Proliferation index (Ki 67)*  *Low (<14%)*  *High (≥14%)* | *272*  *85* | *173.12*  *197.82* | *0.053* |
| *Molecular classes*  *Luminal A*  *Luminal B*  *HER2 enriched*  *Triple Negative* | *165*  *68*  *40*  *57* | *145.39*  *167.35*  *177.15*  *213.34* | ***<0.001*** |

Significant *p* values are in **bold.** Mean rank operated by Mann-Whitey test and Kruskal test. No: Number. FEN1; Flap endonuclease 1. DCIS; Ductal Carcinoma *in Situ*. HER2; Human epidermal growth factor receptor 2.
